# Factors Associated with Time Spent Performing Housework/Childcare by Fathers of Children Aged Under 12 Years: A Cross-Sectional Study in Japan

**DOI:** 10.31372/20200503.1111

**Published:** 2020

**Authors:** Rina Matsubara, Naoko Hikita, Megumi Haruna, Emi Sasagawa, Kaori Yonezawa, Yumi Maeda, Yuka Ikeda

**Affiliations:** a School of Integrated Health Sciences, Faculty of Medicine, The University of Tokyo, Tokyo, Japan; b Department of Midwifery and Women’s Health, Division of Health Sciences and Nursing, Graduate School of Medicine, The University of Tokyo, Tokyo, Japan; c ICT and Media Strategy Group, Digital Innovation Division, Mitsubishi Research Institute, Inc, Japan; d Service Promotion Group, Innovation Service Creation Division, Mitsubishi Research Institute, Inc, Japan

**Keywords:** housework, work–life balance, fathers’ performance of housework, job environment, child

## Abstract

**Objective:** The objective of this study is to determine the factors associated with time spent performing housework/childcare by fathers of children under 12 years of age (preschool and elementary school children) in Japan.

**Methods:** An online survey of employees and employers was conducted in 2017. Of the 7,796 total responses to the survey, those of 621 fathers of children aged 0–12 years were analyzed. Participants were divided into two groups: fathers of children aged 0–6 years, and fathers of children aged 7–12 years. Multiple logistic regression analysis was performed on the items for which there was a significant intergroup difference, setting time spent performing housework as the dependent variable and the survey items as the independent variables.

**Results:** For fathers of children aged 0–6 years, working 40 hours or less per week and commuting for one hour or less were associated with more time spent performing housework/childcare. For fathers of children aged 7–12 years, working less than 40 hours a week, working from home more than once a week, and having spouses with full-time jobs were associated with more time spent performing housework/childcare.

**Conclusion:** This study revealed the factors associated with the time spent by fathers of children under 12 in performing housework/childcare. As several of these factors cannot be altered by the fathers’ efforts alone, companies and society in general must endeavor to improve work styles to better suit the habits and preferences of fathers. This will promote work–life balance and create better family relationships.

## Introduction

Japan’s declining birthrate and aging population have resulted in a recent decreasing trend in the country’s working-age population. This has created various challenges related to, for example, identifying the means of improving productivity, expanding employment opportunities, and creating an environment in which employees can fully demonstrate their motivations and abilities. To address these challenges, the Gender Equality Bureau Cabinet Office (2015a) has proposed several plans with the goal of creating a society in which all individuals, regardless of gender, can fully exercise their individuality and abilities. In addition, the “Act on the Promotion of Female Participation and Career Advancement in the Workplace” was enforced in 2015 (Gender Equality Bureau, Cabinet Office, 2015b). This law is designed to help women who intend to pursue, or are pursuing, a working life to fully realize their individualities and abilities. A notable stipulation in this law is establishing an environment that facilitates a balance between work and family life.

The Ministry of Internal Affairs and Communications’ “Survey on Time Use and Leisure Activities” examined the length of time that married couples with children under 6 years of age spend performing housework/childcare, and found that mothers spend an average of 7 hours and 34 minutes per day, while fathers spend an average of 1 hour and 23 minutes (Bureau of Statistics of the Ministry of Internal Affairs and Communications, 2017). The number of males in Japan who believe that fathers should perform housework/childcare has risen in recent years (Terami & Minami, 2017), and the time fathers spend performing housework/childcare is concurrently increasing (Bureau of Statistics of the Ministry of Internal Affairs and Communications, 2017). However, fathers in the United States, Germany, Sweden, and Norway spend over 3 hours per day performing housework/childcare, indicating that fathers in Japan still spend less time on housework/childcare than their peers in other developed countries (Gender Equality Bureau, Cabinet Office, 2017). Most people cite a lack of time because of work commitments as the reason for being unable to perform housework/childcare (Terami & Minami, 2017). Thus, many women in dual-income households are unable to obtain support from their husbands regarding housework/childcare, and consequently experience the burdens of both their professional work and housework, which is similar to working two shifts a day (Oishi, 2015). In order to balance work and family life among women with children, measures to solve this situation are necessary.

Previous studies have identified several factors related to the time fathers spend performing housework/childcare. Fathers who have shorter commuting times or working hours and those who have many children have been found to spend more time performing housework/childcare (Sasaki, 2018). Moreover, fathers with higher educational attainment than the mothers spend less time performing housework/childcare (Sasaki, 2018). Furthermore, if the fathers live with their parents, or if the fathers or mothers believe that fathers should not perform housework, the fathers’ share of housework is lower. A past study has also indicated that when a couple cooperates in balancing professional work and housework, the father’s share of the housework increases (Kubo, 2009). The traditional perspective in Japan is that men and women should have clearly divided roles (Cabinet Office, Government of Japan, 2018), with a strong belief that men’s primary roles are occupational, which lowers men’s contribution to housework/childcare (Takidai & Kitamiya, 2019).

All the above studies, however, targeted fathers of children aged under 6 years; few studies have targeted fathers of school-age children (Kubo, 2009). Although the time required to perform childcare is expected to decrease as the child ages, as the father plays an important role in the development of a child’s personality, the father’s involvement in childcare is essential (Paquette, 2004). In particular, it is important for late school-age children in the prepubertal stage—a time when children begin to form a perception of the father’s role by observing their own fathers’ behaviors—to have good relationships with their fathers (Maehara & Saito, 2014).

It has been reported that there is a positive relationship between the time fathers spend performing housework/childcare and the likelihood of the couple having a second child (Cabinet Office, Government of Japan, 2018). Additionally, it has also been reported that fathers’ performance of housework/childcare improves mothers’ perceptions of their relationship with the fathers, reduces mothers’ negative feelings regarding being a parent, and increases the fathers’ life satisfaction and development as a parent (Kashiwagi & Wakamatsu, 1994). Therefore, identifying the factors that influence the time fathers spend performing housework/childcare can provide useful information for the development of environments in which fathers more readily perform housework/childcare.

The aim of this study was to identify the factors associated with the time fathers of children aged under 6 years (preschool children) and those of children aged 7–12 years (elementary school children) spend performing housework/childcare. Revealing the factors related to time spent performing housework/childcare in both groups may help identify measures that can improve the working environments of fathers of children of different ages, which may further contribute to increasing fathers’ housework/childcare time.

## Methods

### Study Design and Sample

This cross-sectional study is a part of a larger cooperative research endeavor that was conducted online in September 2017 by the Department of Midwifery and Women’s Health, Division of Health Sciences and Nursing, Graduate School of Medicine, the University of Tokyo, and the Mitsubishi Research Institute, Inc. The target participants of the survey were 10,000 employees or employers, both men and women, aged 20–60 years. The target participants were randomly selected from the 1,200,000 registered, active members of the web-based survey company used for the research. Of the 10,000 individuals approached for participation, a total of 7,796 responded to the web survey, of whom 3,927 were male and 1,878 had children. Of these, 621 males had children under 12 years of age and were selected as the sample for the present study.

### Data Collection

A self-administered online questionnaire comprising 76 items was used for data collection in this study. The items concerned the participants’ socioeconomic characteristics, working environments, lifestyle, physical and mental conditions, spouses’ characteristics, and family environments.

### Variables

The socioeconomic data that were collected concerned each participant’s (and his spouse’s) age, employment type (i.e., “regular employee,” “other” [representing part-time employee, temporary employee, self-employed, or freelancer]), educational attainment (“junior high school or high school,” “vocational school or junior college,” “university,” “graduate school”), and household income (“<2.5 million yen,” “2.5–4.5 million yen,” “4.5–7.0 million yen,” “7.0–10 million yen,” “≥10 million yen”).

The data regarding working environment included working hours over the past 7 days (“≤40 hours,” “≥41 hours”), frequency of working from home (never, more than once a week), commuting time (“<30 minutes,” “30 minutes–1 hour,” “≥1 hour”), history of taking childcare leave (“yes,” “no”), and ambition to obtain a managerial position (“yes,” “neither yes nor no,” “no”).

The participants were asked a selection of questions from the absenteeism and presenteeism questions of the World Health Organization’s Health and Work Performance Questionnaire (Kessler et al., 2003; World Health Organization, 2013), focusing on usual job performance over the past two years. Usual job performance was rated using an 11-point scale ranging from 0 (“worst performance”) to 10 (“top performance”). The K6, which is a psychological distress screening scale developed by Kessler et al. (2002), was used to obtain data regarding the participants’ physical and mental conditions. The K6 comprises six items; the Japanese version was developed by Furukawa et al. (2008). Scores for each item range from 0 to 4 points, with a total score of 0–24 points. Higher scores indicate stronger symptoms of depression and anxiety. Cronbach’s *α* coefficient for the present study was 0.954 among the fathers of children aged less than 6 years, and 0.943 among the fathers of children aged 7–12 years.

The data regarding the participants’ spouses included employment type (“regular employee,” “other” [part-time employee, temporary employee, self-employed, or freelancer]), and hours worked per day (“< eight hours/day,” “≥ eight hours/day”). To determine the family environment, data regarding the following were collected: the youngest child’s age, whether the respondents felt they could easily discuss adjusting housework/childcare burdens with their spouses (“yes”/“no”), whether the respondents were living with their parents (“yes”/“no”), whether they had a person close to them who they could ask to provide assistance regarding housework/childcare (“yes”/“no”), and average time spent performing housework/childcare each weekday (“< 1 hour,” “1 hour,” “2 hours,” “3 hours” […] “14 hours,” “15 hours”).

### Operational Definition

In this study, the length of time spent performing housework/childcare on weekdays was divided into two groups: <2 hours per day and ≥2 hours per day. This criterion was set after referring to data regarding the average time Japanese fathers spend performing housework/childcare (1 hour and 23 minutes) (Bureau of Statistics of the Ministry of Internal Affairs and Communications, 2017). As the response options for time spent performing housework/childcare in this study were “< 1 hour,” “1 hour,” “2 hours,” “3 hours,” etc., we set the cutoff point between “1 hour” and “2 hours.”

### Statistical Analysis

The participants were stratified into two groups according to their children’s age: one group comprised fathers of children aged under 6 years, while the other comprised fathers of children aged 7–12 years. First, we calculated the descriptive statistics for each survey item for the participants who performed <2 hours of housework/childcare and for those who performed ≥2 hours per day, and compared them using Student’s *t*-test for continuous variables and chi-square tests for categorical variables. Then, we performed multiple logistic regression analysis on the items that showed a significant difference (*p* < 0.05), setting time spent performing housework as the dependent variable, and the survey items as the independent variables. Multicollinearity was evaluated using Spearman’s rank correlation coefficient; if the coefficients exceeded |ρ| > 0.5, one of the variables was removed from the multiple logistic regression analysis. Furthermore, we compared the fathers of children aged under 6 years with the fathers of children aged 7–12 years in terms of time spent performing housework/childcare. All data were analyzed using IBM SPSS Statistics 20 for Windows (IBM Corp., Armonk, NY, USA). Two-tailed *p* values of <0.05 were considered to indicate statistical significance.

### Ethical Considerations

This study was approved by the Research Ethics Committee of the Graduate School of Medicine, the University of Tokyo, Japan (12051-(1)). A document describing this study was posted on the home page of the Department of Midwifery and Women’s Health, Division of Health Sciences and Nursing, Graduate School of Medicine, the University of Tokyo.

## Results

Of the 621 male respondents who had children under 12 years of age, 342 were fathers of children aged under 6 years. Of these, 102 (29.8%) spent less than 1 hour performing housework/childcare, 70 (20.5%) spent 1 hour, 86 (25.1%) spent 2 hours, and 84 (24.6%) spent over 3 hours. Thus, 172 (50.3%) fathers performed housework/childcare for less than 2 hours, while 170 (49.7%) performed housework/childcare for 2 hours or more.

Of the overall sample, 279 were fathers of children aged 7–12 years. Of these, 124 (44.4%) spent less than 1 hour performing housework/childcare, 59 (21.1%) spent 1 hour, 50 (17.9%) spent 2 hours, and 46 (16.5%) spent over 3 hours. Thus, 183 (65.6%) fathers performed housework/childcare for less than 2 hours, while 96 (34.4%) performed housework/childcare for 2 hours or more. A significant difference was found when comparing the fathers of children aged under 6 years and those of children aged 7–12 years in terms of the length of time spent performing housework/childcare (*p* < 0.001).

Table 1 shows the results of the bivariate analysis among the fathers of children aged under 6 years. Age (*p* = 0.032), employment type (*p* = 0.038), hours worked within the past 7 days (*p* = 0.002), commuting time (*p* = 0.001), history of taking childcare leave (*p* = 0.031), ambition to obtain a managerial position (*p* = 0.042), usual job performance over the past year or two (*p* = 0.027), youngest child’s age (*p* = 0.036), and ability to easily discuss sharing housework/childcare burdens with one’s spouse (*p* = 0.014) differed significantly between the fathers who spent long periods performing housework/childcare and those who spent short periods.

**Table 1:** Characteristics of Participants with Children Under 6 Years (n = 342)

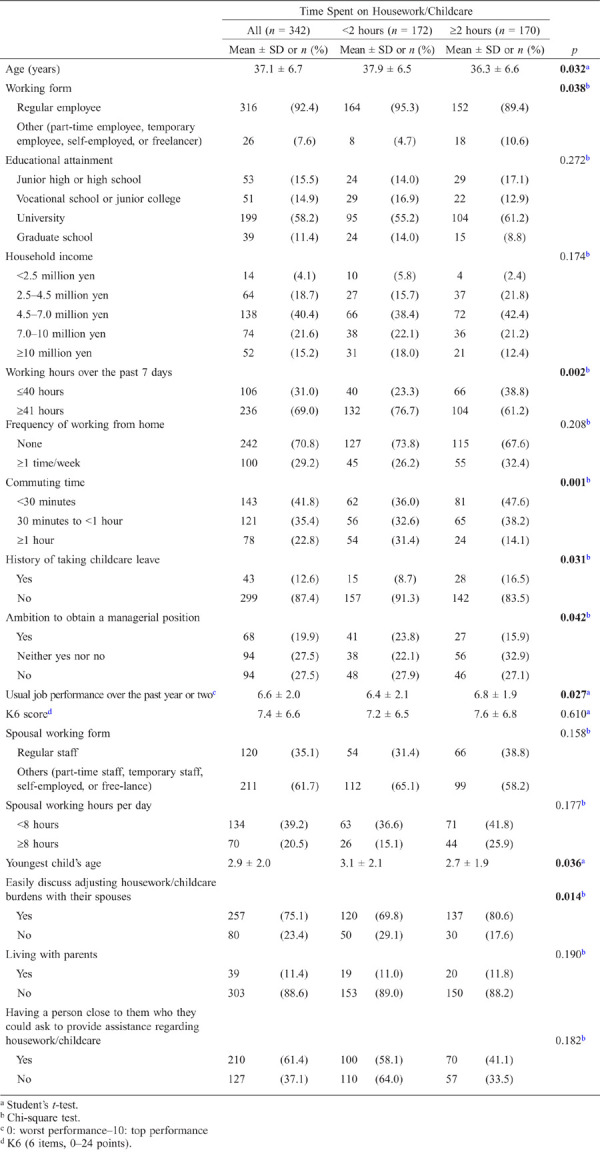

Table 2 shows the results of the bivariate analysis among the fathers of children aged 7–12 years. Age (*p* < 0.001), working hours in the past 7 days (*p* = 0.013), number of days working from home (*p* = 0.001), history of taking childcare leave (*p* = 0.044), K6 score (*p* = 0.010), spouse’s employment type (*p* = 0.001), spouse’s working hours per day (*p* = 0.003), youngest child’s age (*p* = 0.036), and ability to easily discuss sharing the housework/childcare burden with one’s spouse (*p* = 0.036) differed significantly between fathers who spent long periods performing housework/childcare and those who spent short periods.

**Table 2:** Characteristics of Participants with Children Aged 7 to 12 Years (n = 279)

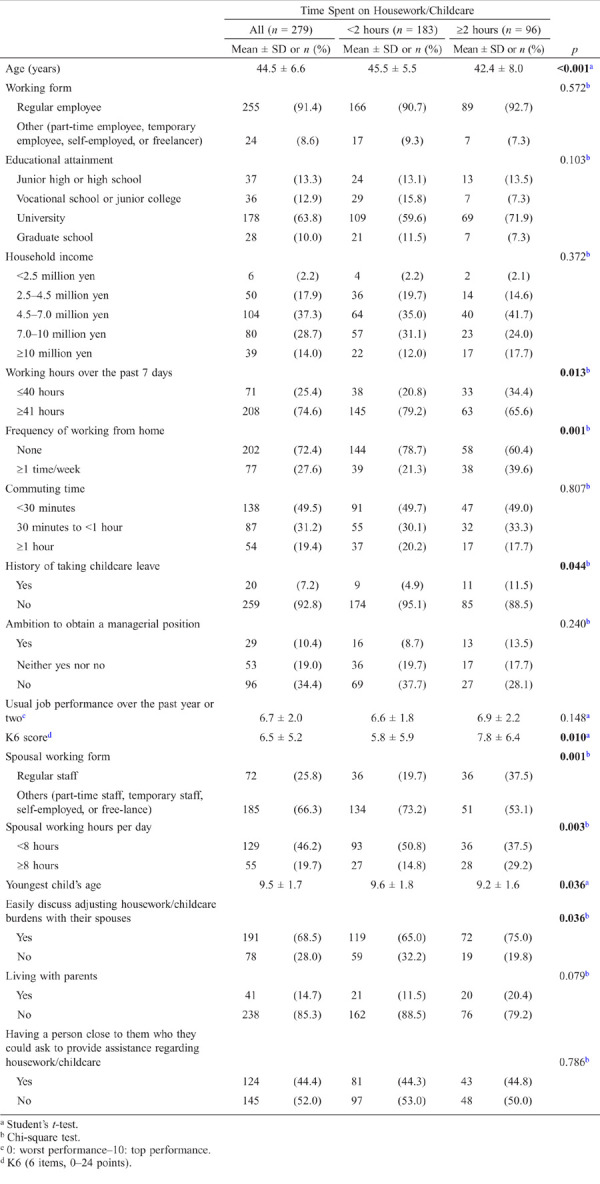

### Factors Associated with the Time Fathers of Children Aged Under 6 Years Spend Performing Housework/Childcare

No multicollinearity was found between the variables; thus, all variables were included as independent variables in the multiple logistic regression analysis. Table 3 shows the results of the multiple logistic regression analysis for factors associated with the time spent in performing housework/childcare by fathers of children aged under six years. Fathers who had worked 40 hours or less in the past 7 days spent more time performing housework/childcare than those who had worked 41 hours or more in the past 7 days (adjusted odds ratio [AOR] = 2.35, 95% confidence interval [CI]: 1.28–4.33). Fathers whose commuting time was less than 30 minutes and those whose commuting time was 30 minutes to 1 hour spent more time performing housework/childcare than those whose commuting time was 1 hour or more (AOR = 3.22, 95% CI: 1.47–7.05; AOR = 2.36, 95% CI: 1.14–4.90, respectively).

**Table 3:** Factors Related to Time Spent on Housework/Childcare Among Fathers with Children Under 6 Years Old (n = 342)

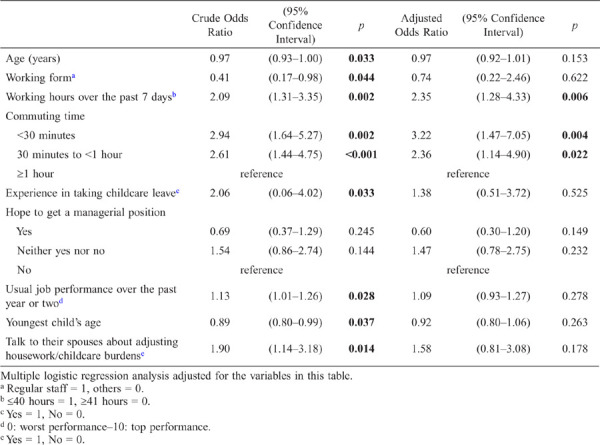

### Factors Associated with the Time Fathers of Children Aged 7–12 Years Spend Performing Housework/Childcare

Multicollinearity was found between spouse’s employment type and daily working hours (*ρ* = 0.59, *p* < 0.001); thus, spouse’s daily working hours was removed as an independent variable from the multiple regression analysis. Table 4 shows the results of the multiple logistic regression analysis for factors associated with the time fathers with children aged 7–12 years spent performing housework/childcare. Fathers who had worked 40 hours or less in the past 7 days spent more time performing housework/childcare than those who had worked 41 hours or more in the past 7 days (AOR = 2.81, 95% CI: 1.48–5.32). Fathers who worked from home more than once per week spent more time performing housework/childcare than those who did not work from home (AOR = 2.17, 95% CI: 1.14–4.16). Finally, fathers whose spouses’ employment type was “regular employee” spent more time performing housework/childcare than those whose spousal employment type was “other” (AOR = 2.51, 95% CI: 1.34–4.69).

**Table 4:** Factors Related to Time Spent on Housework/Childcare Among Fathers with Children Aged 7 to 12 Years Old (n = 279)

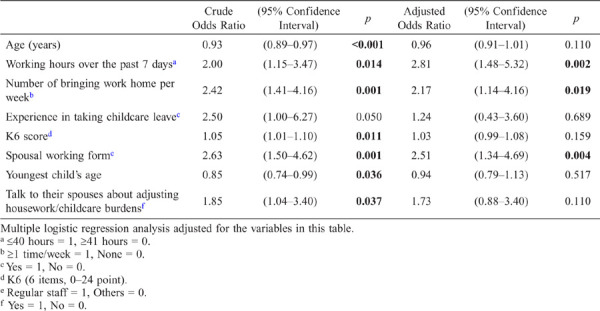

## Discussion

### Comparison of the Fathers of Children Aged Under 6 Years and Those of Children Aged 7–12 Years in Regard to Time Spent Performing Housework/Childcare

Of this study’s sample, 49.7% of the fathers of children aged under 6 years spent over 2 hours performing housework/childcare, whereas this proportion was 34.4% among the fathers of children aged 7–12 years. Compared to the group of fathers of children aged under 6 years, the group of fathers of children aged 7–12 years featured a significantly lower proportion of members who spent more than 2 hours performing housework/childcare. A previous study demonstrated that the time fathers spend performing housework/childcare increases if they have preschool-age children (Matsuda & Suzuki, 2002); therefore, the present results support the results of this previous study. A possible reason for this finding is that, as children’s age increases, childcare becomes less difficult. Moreover, as children’s age increases, fathers’ age also increases, thereby increasing their likelihood of obtaining higher positions in their workplaces, which can shorten the time fathers spend at home.

### Factors Associated with the Time Fathers of Children Aged Under 6 Years Spend Performing Housework/Childcare

In this study, the fathers of children aged under 6 years who had worked 40 hours or less in the past 7 days spent more time performing housework/childcare than those who had worked 41 hours or more in the past 7 days. This result is consistent with that of a previous study, which showed that fathers with longer working hours tend to spend shorter periods providing childcare (Takidai & Kitamiya, 2019). Fathers with shorter working hours have more time outside of work than fathers with longer working hours; therefore, they have more time to perform housework/childcare. Therefore, it is recommended that, after determining employees’ family status, companies provide their employees with information regarding the system for adjusting working hours. In other words, it is important to talk to employees and, if necessary, adjust their work content and working hours so that they can increase the time they spend at home.

Fathers with shorter commuting times performed more housework/childcare than those with longer commuting times. This result is consistent with the “time constraint hypothesis” indicated in a previous study (Sasaki, 2018). Fathers with shorter commuting times are likely to have more time to spend at home than fathers with longer commuting times; thus, it may be easier for the former group to find time to perform housework/childcare. If companies enhance the housing allowance, and/or if fathers adjust their place of work and/or place of residence so that their commuting time is shortened, fathers will have increased time to perform housework/childcare.

As the traditional perception of the division of roles between men and women remains prevalent in Japan (Office for Work-Life Balance, Gender Equality Bureau, Cabinet Office, 2008), it can be difficult for men to take childcare leave, as their bosses are likely to perceive this negatively (Suzuki, 2014). Furthermore, this perception means that it is also difficult to ask coworkers for their cooperation or to discuss one’s own childcare situation and seek moral assistance (Kawaguchi, Matsubara, Iguchi, & Syouji, 2016). Thus, adjusting one’s place of residence to make time for housework/childcare may be challenging. It may be necessary to not only change the perceptions of the father himself, but also to improve awareness in the workplace.

### Factors Associated with the Time Fathers of Children Aged 7–12 Years Spend Performing Housework/Childcare

Among the fathers of children aged 7–12 years, those who had worked 40 hours or less in the past 7 days spent more time performing housework/childcare than those who had worked 41 hours or more over the past 7 days. This result was consistent with the result for fathers of children aged under 6 years, but it is a novel finding for fathers with school-age children. This indicates that, regardless of the children’s age, fathers with shorter working hours (who have more time to spend outside of work than fathers with longer working hours) have more time to perform housework/childcare.

Fathers who worked from home more than once per week spent more time performing housework/childcare than those who did not work from home. This is another novel finding of our study. As fathers who work from home naturally spend more time at home, they can spend more time performing housework/childcare. A previous paper reported that fathers implemented certain measures (within the scope of their individual circumstances) to increase the time available to them to perform childcare/housework (Kawaguchi et al., 2016); working from home might be one such effort. The Ministry of Health, Labor, and Welfare has recommended the introduction of working from home; working at satellite offices (where such offices offer convenience for commuting and working) and home offices; and telework such as mobile work while traveling or visiting customers or in cafés (Ministry of Health, Labor and Welfare, 2018). In this way, providing flexibility to employees regarding the place where they can work (such as by allowing working from home instead of working in the company premises) increases the amount of time they can spend at home. This would increase the time available to perform housework/childcare. It is expected that the number of companies facilitating telework will increase in the future; however, there will still be people who will be unable to work from home; for example, those in the service industry. Therefore, it is necessary to determine the current situation of such employees, as the services they provide play an important role in our daily lives.

In this study, fathers whose spouses were regular employees spent more time performing housework/childcare than those whose spouses’ employment type was “other.” This result is consistent with the “relative resource hypothesis” (Kubo, 2012). The relative resource hypothesis states that, in a couple, the person with fewer resources, such as educational attainment and income, performs most of the housework (Inaba, 1998); the smaller the intercouple difference in regard to educational and income, the more equal the housework-sharing becomes. These results are also consistent with the results of a previous study demonstrating that fathers whose spouses were company employees spent longer hours in parenting than those whose spouses had other job types (Kubo, 2017; Takidai & Kitamiya, 2019). This might be because if the mother is a regular employee, she may have less time to perform housework/childcare, thus requiring the father to spend more time in performing housework/childcare.

### Strengths and Limitations

This is the first study to reveal the factors associated with the time spent in performing housework/childcare by fathers of children aged under 6 years and those of children aged 7–12 years. As the child grows older, the time and effort required to perform childcare decreases; meanwhile, the father’s age also increases, along with (possibly) his social status. Thus, revealing the factors associated with the time spent performing housework/childcare among not only fathers of preschool-age children, but also fathers of elementary school-age children can provide suggestions regarding how fathers’ work environments could be adjusted depending on their children’s ages.

Despite this strength, there are several limitations to this study. First, this study utilized a self-administered questionnaire survey for people registered at an Internet survey company. The response rate of this study was approximately 78%, meaning we did not obtain responses from all of the randomly selected individuals. This may have negatively impacted the generalizability of the present findings. In addition, it is possible that the respondents exerted minimal effort when responding to the questionnaire (Miura & Kobayashi, 2016). Second, the questionnaire comprised 76 items, further increasing the possibility that the respondents exercised minimal effort when responding to some items. Third, the meaning of housework/childcare may differ across respondents, which may have influenced the participants’ answers. Fourth, we did not ask the participants whether they were single parents, and whether they had one child or multiple young children. Thus, the number of children was not taken into account. If they were single parents or had more than one child, the time spent performing housework/childcare could possibly increase. Further studies addressing some of the limitations of this study are needed.

## Conclusion

This study revealed the factors associated with the time spent in performing housework/childcare by fathers of children under 12 years of age. The number of working hours was found to be related to the number of hours spent by fathers in performing housework, regardless of their children’s age. Commute length was associated with the number of hours fathers of children aged 0–6 years spent performing housework; meanwhile, working from home and spousal employment status were related to the number of hours fathers of children aged 7–12 years spent performing housework. As these factors cannot be altered by fathers’ individual efforts, companies as well as society at large must make efforts to improve working arrangements to create more suitable circumstances for parents. This will promote work–life balance and foster better family relationships.

## Acknowledgments

We are grateful to all the participants who answered the online questionnaire.

## Declaration of Conflicting Interests

The authors declared no potential conflicts of interest concerning the research, authorship, or publication of this article.
